# CNCC: an analysis tool to determine genome-wide DNA break end structure at single-nucleotide resolution

**DOI:** 10.1186/s12864-019-6436-0

**Published:** 2020-01-08

**Authors:** Karol Szlachta, Heather M. Raimer, Laurey D. Comeau, Yuh-Hwa Wang

**Affiliations:** Department of Biochemistry and Molecular Genetics, University of Virginia, Charlottesville, Virginia 22903-0733 USA

**Keywords:** DNA double-stranded breaks, Break end type, DNA end resection, Etoposide treatment, Topoisomerase II

## Abstract

**Background:**

DNA double-stranded breaks (DSBs) are potentially deleterious events in a cell. The end structures (blunt, 3′- and 5′-overhangs) at DSB sites contribute to the fate of their repair and provide critical information concerning the consequences of the damage. Therefore, there has been a recent eruption of DNA break mapping and sequencing methods that aim to map at single-nucleotide resolution where breaks are generated genome-wide. These methods provide high resolution data for the location of DSBs, which can encode the type of end-structure present at these breaks. However, genome-wide analysis of the resulting end structures has not been investigated following these sequencing methods.

**Results:**

To address this analysis gap, we develop the use of a coverage-normalized cross correlation analysis (CNCC) to process the high-precision genome-wide break mapping data, and determine genome-wide break end structure distributions at single-nucleotide resolution. We take advantage of the single-nucleotide position and the knowledge of strandness from every mapped break to analyze the relative shifts between positive and negative strand encoded break nucleotides. By applying CNCC we can identify the most abundant end structures captured by a break mapping technique, and further can make comparisons between different samples and treatments. We validate our analysis with restriction enzyme digestions of genomic DNA and establish the sensitivity of the analysis using end structures that only exist as a minor fraction of total breaks. Finally, we demonstrate the versatility of our analysis by applying CNCC to the breaks resulting after treatment with etoposide and study the variety of resulting end structures.

**Conclusion:**

For the first time, on a genome-wide scale, our analysis revealed the increase in the 5′ to 3′ end resection following etoposide treatment, and the global progression of the resection. Furthermore, our method distinguished the change in the pattern of DSB end structure with increasing doses of the drug. The ability of this method to determine DNA break end structures without a priori knowledge of break sequences or genomic position should have broad applications in understanding genome instability.

## Background

DNA double-stranded breaks (DSBs) are one of the most dangerous types of damage that occur in cells, and when unrepaired or illegitimately repaired, DSBs can be cytotoxic or cause genome instability. Multiple pathways to repair these lesions exist, and DNA end structure at the site of the break is one factor determining which pathway is used for repair [1–3]. Homologous recombination (HR) is dependent on the template provided by homologous sequences to repair DSBs, and requires extensive resection of the ends on both sides of the DSB, resulting in extensive single stranded 3′ overhangs. Meanwhile, non-homologous end joining (NHEJ) canonically ligates the two DNA strands with limited end-processing. When a wide range of endogenous and exogenous conditions generate DSBs, different end structures are produced and then affect the efficiency, timing, kinetics, and accuracy of repair at the break sites [4, 5].

The importance of understanding DSBs and their repair prompted the recent eruption of experimental techniques to precisely map/sequence DSBs [6–11]. Each DSB generates two distinct DNA ends and these methods captured these ends which encode the genomic location of the break. Existing studies have primarily used this DSB location data to evaluate breaks occurring at specific loci or subsets of the genome, as opposed to identifying the types of break end structures globally at single-nucleotide resolution. We leveraged that break mapping data provides coverage information on both positive and negative strands for each individual mapped break in the entire genome. Therefore, using a coverage-normalized cross correlation (CNCC) between the coverage on the positive and negative strands, the type of end structures at the breaks (blunt, 3′- and 5′-overhangs) can be revealed at single-nucleotide resolution. Cross correlation analysis allows for patterns to be identified within noisy and sometimes sparse data. Applying CNCC to break mapping and sequencing data means that the genome-wide composition of DSB end structures can be retrieved from data generated by these various methods. Previous methods that have studied the impact of DSB end structures have primarily relied upon either a few specific loci or DNA fragments in an in vitro extract experiment. Therefore, there is a large gap in our understanding of the global impacts of end structures and the repair that follows.

To address both the analysis gap for the break mapping data and the impact of genomic stress on DNA end structures, we develop the use of CNCC analysis. Here, we present the utility of our CNCC method, on a genome-wide level, to distinguish, using multiple restriction enzymes, the three major DNA end structures: blunt-ended, 3′-overhangs, and 5′-overhangs. Additionally, we test the sensitivity of our method to detect an end structure that only represents a small fraction of the acquired break data. Finally, we demonstrate the global endogenous DSB end structure landscape of a non-malignant cell line and the subsequent changes upon treatment with etoposide, a chemotherapeutic drug. This change leads to an increase in 5′ to 3′ end resection products following treatment, which is dose dependent. Overall, our analysis can provide a global view of what type of end structures occur upon DNA damage, and compare trends of damage types resulting from different drug treatments and in different regions of the genome.

## Results

### Overview of end-structure determination by coverage-normalized cross correlation

Each DSB generates two distinct DNA ends, representing each side of the break. Those ends can be captured by DNA break mapping and sequencing methods as two distinct DNA fragments, and provide coverage information on the positive and negative strands for individual breaks. This information can then be used to reveal the end structure of these captured, broken ends. In the genome-wide break mapping protocol that we adapted from the DSBCapture methods [7], the initial end processing by filling in 5′ overhangs and trimming 3′ overhangs, is an essential step that allows downstream analysis to distinguish the two classes of overhangs (see “Methods”). Next, the ligation of the P5 adaptor to the blunted ends captures each of the DSB ends; this allows for the downstream identification of the DSB-proximal nucleotide as the most 5′ nt of read 1 of the sequenced pair for each side of the break (Additional file 1: Figure S1).

**Fig. 1:**
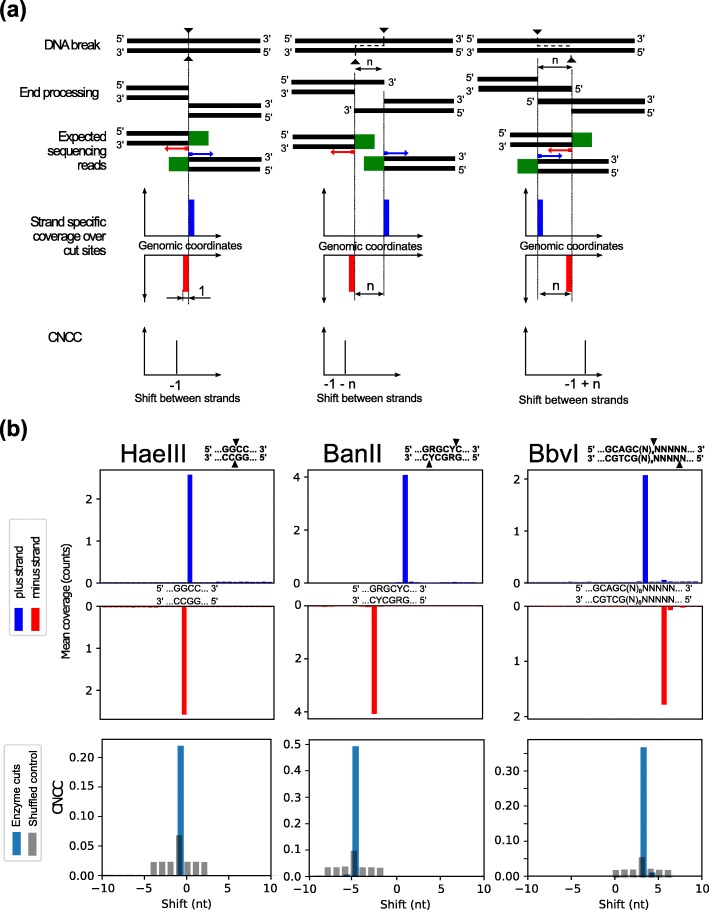
Determination of DSB end structures by CNCC. **a** Outline of the CNCC analysis for three types of breaks: blunt end (left), 3′ end overhang (middle) or 5′ end overhang (right). Each break produces two DNA ends and each of those are processed, 3′ overhangs trimmed and 5′ overhangs filled in (“Methods”), ligated to a sequencing adaptor (green rectangles), and sequenced. These reads result in distinctive patterns of coverage on both positive (blue) and negative strands (red) (fourth row). Conducting our genome-wide CNCC analysis between coverage on positive and negative strands then reveals a shift that is characteristic of the type of DSB end structure (fifth row). **b** CNCC analysis of DNA breaks caused by HaeIII, BanII and BbvI restriction enzyme cleavage (left, middle, and right column, respectively). The mean read coverage over the indicated enzyme motifs presents precise location of mapped reads (top two rows), and genome-wide CNCC spikes (bottom row, blue) at − 1 for HaeIII, − 5 for BanII, and + 3 for BbvI exactly reflect the expected end structures, with shuffled controls in gray. CNCC analysis was employed using all sequencing data, and was not limited to reads found at enzyme motifs

To analyze the types of break end structures on a genome-wide scale, we developed an unbiased analysis approach that leverages the single-nucleotide resolution of every captured break on the positive and negative strands to calculate the most abundant relative shifts between these two groups of signals (we termed the analysis “Coverage-Normalized Cross Correlation, CNCC”). These relative shifts reveal the genome-wide composition of DSB end types at single-nucleotide resolution, without the explicit knowledge of which reads come from any single DSB site or of sequence composition at DSB sites. CNCC is a correlation between two different signals calculated for a range of relative shifts between them and normalized by the total break coverage of a given sample. Therefore, in our analysis, the relative distance between a pair of genomic positions on positive and negative strand codes what type of DSB end-structure was generated. Blunt end breaks have a characteristic shift of − 1, 3′ overhangs have shift values less than − 1, while 5′ overhangs have values greater than − 1 (Fig. 1a).

The advantage of using a cross correlation-based analysis comes from the ability to distinguish patterns within data that may have a high degree of noise and background signal. It allows for the most prominent end type patterns to be extracted from the genome-wide break mapping data we analyze. The CNCC analysis computes the cross correlation for each mapped break position on the positive and negative strand for a defined range of shifts, and then combines these values across the genome. Therefore, the analysis output demonstrates a composite profile of the end type signatures for all breaks mapped in the given data set, and dominant end structures can be identified. In this way the analysis is blind in regards to any treatment or expectation for end-structure. Furthermore, downstream analysis can be used to identify which mapped break signals are contributing to any given end-structure signal.

### Validation of CNCC end-structure determination

To determine the ability of our approach to successfully distinguish the three different types of end structures, we first evaluated the DNA breaks generated by restriction enzyme cleavage. Digestion of isolated genomic DNA from non-malignant GM13069 lymphoblasts by BanII, BbvI, and HaeIII enzymes respectively produces 3′ overhangs, 5′ overhangs and blunt-end breaks. Break mapping and sequencing of the enzyme-digested samples resulted in 2.0 million, 2.3 million, and 3.9 million enzyme cut sites with reads, and with reads at cut sites accounting for 90, 61, and 97% of total reads, respectively (Additional file 1: Table S1). Each library was sequenced to at least 15 million reads (Additional file 1: Table S2), and the mean read coverage over cut-site regions showed a clear enrichment of breaks at cut sites with minimal background (Fig. 1b, top two rows). The application of genome-wide CNCC analysis, using all mapped DNA ends for each enzyme-digested sample (Additional file 1: Table S2), demonstrated that restriction enzyme shifts of CNCC spike at − 1 for HaeIII, − 5 for BanII, and + 3 for BbvI (Fig. 1b, bottom) precisely corresponded to the expected DNA end types: blunt end, 4-nt 3′ overhang, and 4-nt 5′ overhang, accordingly. Additionally, comparing technical replicates of the BbvI digestion experiment, showed near perfect reproducibility of both genomic coverage and restriction enzyme shift spikes of CNCC between experiments (Additional file 1: Figure S2). To further validate our method, we analyzed published data from an EcoRV digestion in HeLa cells [7], and detected an enzyme spike of CNCC at − 1 shift which corresponded exactly with the expected blunt end structure generated by EcoRV (Additional file 1: Figure S3). Altogether, these results suggest that our CNCC analysis is both robust in its ability to accurately distinguish the three different major end-structures and is highly reproducible.

While validating the accuracy of end structure detection by CNCC, the appropriate control for this analysis was also investigated. To establish a meaningful control, first a simple random shuffle was implemented. However, considering that the human genome is on the order of 3 × 10^9^ nt, performing a simple random shuffle over this large region produced nearly nonexistent background levels. This result comes from both the size of the shuffling region being so large and the disruption of break cluster sites by the unrestrained random shuffle. Therefore, to create a more stringent control, we introduced much smaller perturbations to the signal to conserve the overall composition of the break intensities and clustering. First, we shuffled single-nucleotide coverage intensities between regions that originally had non-zero coverage, therefore maintaining break clustering. Second, to each genomic position with non-zero break coverage, we added a random value to wiggle the position, and defined the range available for this random value in order to control the size of the region in which the signal is shuffled. A series of different wiggle ranges were tested on the HaeIII data to establish which would give the most meaningful control (Additional file 1: Figure S4a). We determined that the magnitude of the wiggle directly corresponds to the maximum spike range, which the control is needed for. This allows for a meaningful shuffle and also minimizes the size of the perturbation to obtain an appropriately stringent control. For the restriction enzyme digestion experiments, because a single end-structure species is expected the maximum spike range to be controlled for is 1, we then used a wiggle ranging from + 2 to − 2 (0, no shift, excluded) to generate the shuffled control (Fig. 1b, bottom, Additional file 1: Figures S2 and S3).

### Testing CNCC sensitivity

**Fig. 2:**
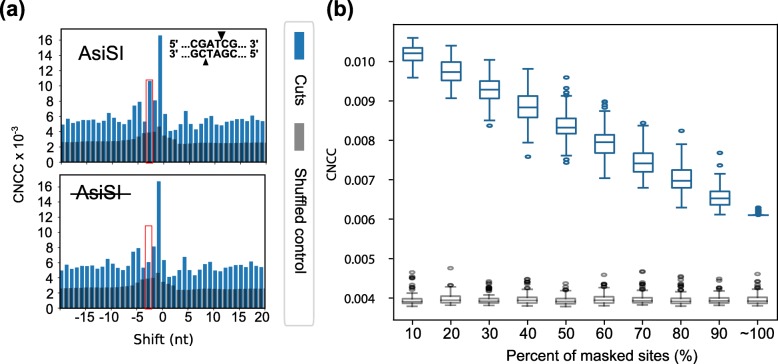
Analysis of CNCC end structure determination sensitivity. **a** CNCC analysis of DSBs generated by AsiSI limited digestion (top). When all AsiSI cut sites (0.03% of total reads) are masked, CNCC at − 3 shift (marked by red rectangles) drops to the average level (bottom). **b** The CNCC signal at − 3 shift (blue) with an increasing percentage of cut sites masked shows a concordant decrease in the CNCC signal at − 3 shift, while the corresponding shuffled control remains constant (gray). Box represents 25 and 75%, and median is marked with the bar within the box. Whiskers depict 5 and 95%, and outliers are marked with ellipses

For CNCC to be broadly applicable to investigate biologically relevant questions, it will need to be able to determine break end structures from samples that have heterogeneous overhang lengths and where any given overhang may exist as a much lower proportion of the total breaks. To assess the sensitivity of CNCC analysis to detect the end structure of DNA breaks present at a low fraction of the total breaks, we processed published break mapping data from a limited AsiSI digestion in U2OS cells [7]. In this system, only a fraction of the total reads (0.03%, ~ 30,000 reads) were generated from the AsiSI digestion, compared to nearly 61–97% of reads attributed to enzyme cleavage in our previous analyses (Fig. 1b). AsiSI digestion produces a 2-nt 3′ overhang DNA end structure, which corresponds to a shift spike for CNCC at − 3, and our analysis indeed detected this expected spike (Fig. 2a, top) along with a variety of other endogenous break signals. When all reads located at AsiSI cut sites were found and removed from the analysis, we observed a concordant drop in the spike of CNCC at − 3, without altering any other spikes (Fig. 2a, bottom). This signal drop demonstrated both the accuracy of CNCC since removing AsiSI sites only affected the spike associated with its end structure and the sensitivity of the analysis to this break signature that accounted for only 0.03% of total break mapping data.

Next, sensitivity was further probed to determine a detection limit necessary for a change in the frequency of an individual break end structure to be identified by our method. Therefore, CNCC analysis was performed while incrementally masking a random 10% of the AsiSI cut sites, and performing 100 iterations for each 10% increment of random site masking. Results showed that while the shuffled control level remained unchanged (grey), a gradual, linear decrease of CNCC for the − 3 spike was observed (blue) (Fig. 2b). This exercise clearly demonstrates the high sensitivity of CNCC to detect a break species that exist only as a minimal contributor to total break signal and further detect even small changes in the composition of the DNA break end structure within a larger pool of break signals.

### Investigating the end structures of endogenous and etoposide-induced breaks

In the AsiSI sensitivity analysis (Fig. 2a), along with the expected − 3 spike attributed by the enzyme, we also observed a large break spike of CNCC at − 1 shift, suggesting a high abundance of blunt end breaks among the endogenous break profile. To determine if this spike was specific to U2OS cells or a more broadly occurring endogenous break species, we analyzed the CNCC break profile of untreated non-malignant lymphoblast cells, GM13069, and revealed a similar pattern (Additional file 1: Figure S5a). Further, to identify location of the endogenous breaks, the genomic regions containing breaks were annotated and the distribution of breaks was determined. This analysis demonstrated that endogenous breaks were enriched at promoters and transcription start sites (TSS), with 48% of the total break density localized to these regions following normalization to account for total region sizes (Additional file 1: Figure S5b). Enrichment in these two genomic regions agrees with previous break mapping data [7, 9].

**Fig. 3:**
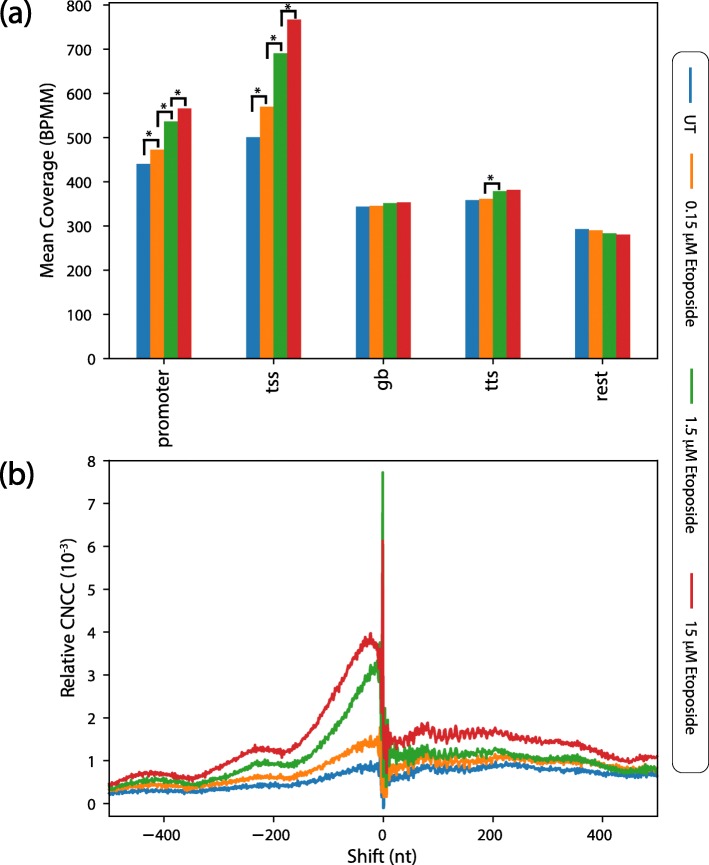
Determination of break density change and end structure response following etoposide treatment. Results demonstrate that inhibition of TOP2 with etoposide increases break densities at promoters and TSSs, reveals increased genome-wide 3′-overhang end structures, and displays the progression of 5′ to 3′ resection. **a** Total break density for two biological replicates in each annotated genomic region normalized to region size for all etoposide treatments (mean coverage as breaks per megabase per million (BPMM)). Increases in break density seen in the promoter and TSS are significant for each increasing etoposide treatment step (*p* < 2.2 × 10^− 16^ is denoted as *). Significance was determined by Kruskal-Wallis test for each region, and followed up with Dunn tests using the Benjamini-Hochberg method of correction. **b** Treatment breaks of relative CNCC for etoposide-treated cells over a 1000-nt shift. Data represents the merge of two biological replicates for each treatment (Additional file 1: Table S2 and Figure S9)

To demonstrate the applicability of CNCC to broader research interests, we employ our CNCC analysis to examine end structures at these two genomic regions. The observed enrichment of reads at TSSs and promoters prompted us to examine the contribution of topoisomerase II (TOP2), which is known to act at promoters and TSSs [12–14]. To explore TOP2-mediated breaks, we treated GM13069 cells with three concentrations of etoposide, an inhibitor that prevents the re-ligation activity of TOP2, resulting in a covalently bound protein-DNA cleavage complex. Upon etoposide treatment, there was a dose-dependent increase in cell death examined by propidium iodide staining of the cells and flow cytometry analysis (Additional file 1: Figure S6). The genome-wide break mapping and sequencing was performed with two biological replicates of etoposide-treated samples. Pearson correlations between genome-wide read coverage of DSBs for each treatment showed a strong reproducibility between each biological replicate (*r* = 0.86–0.98, (Additional file 1: Figure S7). Further, there was a concordant and significant dose-dependent increase in break density at promoters and TSSs (*p* < 2.2 × 10^− 16^, Kruskal-Wallis with Dunn test) (Fig. 3a), while all other regions lacked this increase, which is consistent with known locations of TOP2 activity. This increase of breaks at TSSs and promoters following etoposide treatment has been previously suggested [15–20], and our genome-wide analysis directly confirms this change in break occurrence under etoposide treatment.

Next, we applied the CNCC analysis to the two biological replicates of etoposide-treated samples separately (Additional file 1: Figure S8). Although there was slight variability between the replicates, the general shape and trend for each etoposide dose remained consistent, with the most variability being seen in the case of 15 μM which may be due to the high dosage. We also observed that the CNCC analysis of the etoposide-treated samples displayed a much broader range of shifts. To establish an appropriate shuffled control for these samples, the 15 μM etoposide treated sample was used to test the same series of wiggle ranges as was done for HaeIII-digested sample (Additional file 1: Figure S4b). A wiggle magnitude of + 2000 to − 2000 (0, no shift, excluded) was identified to be meaningful and was implemented, because the maximum spike range to be controlled for is much larger (Additional file 1: Figure S8).

The biological replicates were merged, CNCC analysis was applied, and the respective shuffled controls for each treatment were generated (Additional file 1: Figure S9a). To properly compare treatments, the median value of the shuffled control for each treatment was subtracted from the CNCC values of the corresponding treatment, to generate a “relative CNCC” (Fig. 3b and Additional file 1: Figure S9b). The relative CNCC signals of etoposide-treated samples showed that with increasing etoposide concentrations, there was an increase in the generation of CNCC spikes over a broad range of negative shifts (Fig. 3b). This broad range of spikes, which have shift values less than − 1, indicates the generation of 3′ overhang ends, and the shape of the CNCC spikes suggests a resection gradient, with a maximum length of approximately 165 nts. TOP2 does not have precise recognition sequences [12, 21, 22], therefore, the display of the 5′ to 3′ end resection globally by the CNCC analysis without knowing specifically targeted sequences or regions demonstrates the valuable utility of CNCC. This 5′ to 3′ resection is likely the result of TOP2 cleavage complexes being removed by MRE11 [5, 23–25] or TDP2 [26–29] and further processed by downstream pathways. Moreover, the 1.5 μM treatment revealed a peak of CNCC resection signature between 4 and 28 nts (determined by 90% of maximum CNCC value in the resection range), representing the most common resected 3′ overhang end structure following this dose of etoposide treatment. Interestingly, the peak of the CNCC resection signature observed for 15 μM etoposide treatment was 5–45 nts, demonstrating that the length of resection increased with higher etoposide treatment. This suggests that our analysis is capable of distinguishing even these fine differences in the end structure distributions between treatments.

## Discussion

While cross correlation analyses have been previously applied to ChIP-seq data [30–32], this is the first time, to our knowledge, this analysis approach has been applied to genome-wide break mapping/sequencing data. We took advantage of the ability of cross correlation analysis to identify patterns in a noisy background, and combined this with the single nucleotide resolution of the break mapping/sequencing data to analyze DNA end structures following damage. We demonstrated the ability of our method of CNCC analysis to determine the genome-wide end-structure distribution of DNA DSBs at single-nucleotide resolution. Our analysis tool has proven to work for both induced break systems (sequence-specific breaks by restriction enzymes and etoposide-induced breaks) to capture the resultant break end structure landscape of the cell. Additionally, for the first time on a genome-wide scale, our method revealed the increase in the 5′ to 3′ end resection following etoposide treatment, and more importantly, the global progression of the resection. The change in the extent of resection could indicate which pathways are being used to repair the DSBs. The difference in DNA end structure at the site of break and the extent of resection in part dictates repair pathway choice between NHEJ, HR, and other pathways such as microhomology-mediated end joining (MMEJ) and single-strand annealing (SSA) [2, 3, 33–36]. While little to no resection and end-processing is supportive of NHEJ, short resection facilitates a shift towards MMEJ, but a long resection drives towards HR or SSA [2]. Further investigation into different repair pathways, or specific proteins in these pathways, can benefit from including mapping genome-wide breaks and coupling with our CNCC analysis to determine the impacts on the repair of various endogenous and induced breaks.

Recently, Gittens et al. developed a method to specifically map genome-wide TOP2 cleavage complexes at single-nucleotide resolution [37]. The mechanism of TOP2 cleavage results in a transient 4-nt 5′ overhang with the TOP2 cleavage complex attached on the 5′ ends [38]. Gittens et al. first applied P7 adaptors to sonication-generated ends, and then utilized TDP2 to remove the TOP2 cleavage complex from the 5′ end prior to the fill-in reaction and P5 adaptor ligation. Therefore, the TOP2 cleavage complex position is encoded by the most 5′-nt of read 1. We applied our CNCC analysis to this single nucleotide data and revealed a spike of CNCC at + 3, as expected (Additional file 1: Figure S10). The + 3 shift spike reveals the 4-nt 5′ overhang of the transient double strand breaks generated by the initial TOP2 cleavage. Again, this demonstrates the power of CNCC analysis to determine end structure without a priori knowledge of break sequence or location. Furthermore, the + 3 shift spike supports that the series of negative shift values that we observed upon the increase of etoposide in our CNCC analysis are a result of the resection of persistent breaks, in part due to the presence of TOP2 cleavage complexes (Fig. 3b).

In addition to the ability to determine end structures at DSBs, the CNCC analysis of broken ends allows for identification of consensus sequences (if they exist) located at the breaks. Using the break data from the BanII digestion, we identified pairs of DSB coverage containing at least two reads, which displayed the spike on the negative strand located 4 nts upstream from the spike on the positive strand (*n* = 718,163) (example genomic sites, Additional file 1: Figure S11a). By performing a motif analysis of these read pair sites, we can recapitulate the BanII consensus sequence (Additional file 1: Figure S11b). This analysis demonstrates that the knowledge of end structure from CNCC analysis can be further used to understand potential sequence motifs associated with identified end structures.

Previously break mapping and sequencing studies (DSBCapture and ENDseq) [7, 8] have used AsiSI digestion to demonstrate their ability to detect break ends. However, they did not carry out analysis to identify and distinguish the type of end structures genome-wide. By applying the CNCC analysis to the AsiSI data derived from DSBCapture [7], we were able to demonstrate both the presence of the AsiSI-induced breaks and determine computationally that a 3′ overhang is generated using the genome-wide data. Further, CNCC can reach these conclusions without any prior knowledge of the expected end type or break location (Fig. 2). The ability of the CNCC analysis to determine the 3′ overhang, 5′ overhang, or blunt end nature of breaks is dependent on the differential processing of 3′ and 5′ overhangs into blunt ends by trimming and filling in, respectively, prior to first adaptor ligation. Methods that do not differentially process these two overhang species will not be able to distinguish them. Therefore, break mapping and sequencing efforts that intend to investigate the impact of the type of end structures in their study system, require the proper processing of break ends. These studies can then be benefitted from the power of CNCC to provide a meaningful analysis.

## Conclusions

Overall, the ability of CNCC to determine DNA break end structures without a priori knowledge of the break sequences or genomic locations can lend itself to multiple analyses, provided the end-structure or break species under study is appropriately sampled in the sequencing data. Our analysis tool can be applied to genome-wide DSB sequence mapping datasets over a broad range of treatment conditions and across cell types to better understand the impact and specificity of treatments on generating breaks, and to investigate the fate of broken ends and proteins that repair them. In the future, CNCC analysis can be implemented in studies to further understand DNA repair mechanisms and genome stability.

## Methods

### Cell culture and treatments

GM13069 cells, a human lymphoid cell line derived from a normal individual (Coriell Institute, catalog ID GM13069), were grown in RPMI 1640 medium (Gibco 11,875) supplemented with 10% fetal bovine serum and plated at 2 × 10^6^ cells per 100 mm cell culture dish. Cells were treated 18 h later with etoposide (Sigma E1383) at 0.15 μM, 1.5 μM, or 15 μM for 24 h. Cells and cell culture medium were then collected by centrifugation at 4 °C and washed twice with cold PBS containing the treatment dose of etoposide. After washes, cells were divided into two equal aliquots. For cell viability assay, one aliquot of cells was resuspended in 2 ng/mL propidium iodide for flow cytometry analysis using a FACSCalibur flow cytometer (BD Biosciences). For breakpoint detection, genomic DNA was purified from the other aliquot of cells by lysing cells in 50 mM Tris. Cl (pH 8.0); 100 mM EDTA; 100 mM NaCl; 1% SDS; 1 mg/mL Proteinase K for 3 h at 55 °C followed by organic extraction purification and ethanol precipitation. Precautions such as gentle pipetting with wide-opening pipette tips to avoid shredding DNA were taken to avoid introduction of DNA breaks during purification.

### Genome-wide break mapping and sequencing

Detection of DSBs was performed as described [7] with modifications. Briefly, genomic DNA from etoposide treated cells or restriction enzyme-digested DNA were subjected to end-blunting reactions with T4 DNA polymerase, Klenow fragment of DNA Polymerase I, and T4 Polynucleotide kinase. During the reaction (Fig. 1), two blunted ends of each break will stay as blunted; two break ends with 3′ overhangs are trimmed by the 3′ to 5′ exonuclease activity of the polymerases to generate two blunted ends with a separation based on the reference sequence; two break ends with 5′ overhangs are filled-in by the 5′ to 3′ polymerase activity of the polymerases to generate two blunted ends with a overlap based on the reference sequence. The CNCC analysis utilizes the separation or overlap of the two ends to distinguish these three end structures. The end-blunting reactions are followed by A-tailing reactions and Illumina adaptor P5 ligation to broken DNA ends. Excess adaptor was removed and then DNA was fragmented by sonication, and subsequently ligated to Illumina adaptor P7. The libraries were amplified by PCR for 15 cycles. Prepared libraries were then subjected to whole-genome 75 bp paired-end sequencing by the Illumina NextSeq 500 platform.

### Sequencing data analysis

Sequencing reads were aligned to the human genome (GRCh38/hg38) with bowtie2 (v.2.3.0) aligner running in high sensitivity mode (*−−very-sensitive,* critical program options are given in parentheses). Restriction on the fragment length from 100 nt to 2000 nt (*−X 2000 −I 100* options) was imposed. Following alignment, the unmapped, non-primary, supplementary and low-quality reads were filtered out with samtools view (v. 1.7) (*−F 2820*). Further, to ensure reads from independent events, PCR duplicates were marked using picard-tools (v. 1.95) MarkDuplicates. Finally, the read 1 s (*−f 67*) from non-duplicated reads (*−F 1024*) were filtered with samtools view and saved for downstream analysis. Further downstream analysis was performed with BEDtools (v. 2.26.0). Data were visualized in python (v. 3.5.2) with libraries: pandas (v. 0.22.0), numpy (v. 1.13.3), and matplotlib.pyplot (v. 2.0.2). For Fig. 3a, Genomic regions were defined as follows: Promoter region ranging from − 1 knt to − 250 nt of the TSS; TSS region ranging ±250 nt from the TSS; Transcription termination site (TTS) region ranging ±250 nt from the TTS; Gene body region ranging from + 250 nt of TSS to − 250 nt of the TTS; Intergenic region is the rest of the genome, not belonging to any of the four regions detailed above.

### Coverage-normalized cross correlation

First, genome-wide coverage was calculated with bedtools genomecov for each strand separately (*−strand +/−*) using only the 5′ end (*− 5* option) of read 1 of non-duplicated reads (see aligning procedure described above). Genome-wide coverage files were saved in dz. format (*−dz* option). These coverage output files were then read into Jupyter notebooks with python (v. 3.5.2) and used pandas (v. 0.22.0) data frames to implement our coverage-normalized cross correlation calculations (with numpy.dot function), at which time coverage over the centromeres (defined by GRCh38/hg38 ideogram data, based from g-banding) was masked (discarded) prior to final calculation.

Coverage-normalized cross correlation (CNCC) is a cross correlation that is further normalized based on overall coverage as to allow for meaningful comparison between different biological samples. CNCC as such is defined as:

$$ CNCC(t)=\frac{\sum_iy(i)x\left(i-t\right)}{\sqrt{\sum_i{x}^2(i){\sum}_i{y}^2(i)}} $$

where: *x(i)* is DNA break coverage on the positive strand at position *i*, *y(i)* is DNA break coverage on the negative strand at position *i,* and *t* is the shift distance of interest. (Here we are using only the most 5′ nt of read 1 as it exactly maps DNA break position).

Modified, two-step shuffled control was calculated by using the coverage output files from above then implementing both a wiggle of position using random wiggle assignment (with numpy.random.randint) and a shuffling of the coverage values between positions (with numpy.random.permutation) for each sample. Then, CNCC, as defined above, was calculated for the minimally perturbed data to generate the stringent shuffled control. For the restriction enzyme digested samples, we applied a wiggle value ranging from + 2 to − 2 (0 excluded). For the etoposide-treated samples, we applied a wiggle value ranging from + 2000 to − 2000 (0 excluded) (see main text for the discussion on these shuffled controls).

### Sensitivity of CNCC analysis

Published break mapping data from a limited AsiSI digestion in U2OS cells [7] was aligned and processed by the CNCC analysis. The AsiSI enzyme was fused to an estrogen receptor ligand binding domain, and when cells were treated with 4-hydroxytamoxifen, the enzyme was transported to the nucleus where it then generated DSBs at loci containing its consensus sequence. This resulted in a limited digestion where reads from AsiSI cleavage (mapped to the consensus sequence) were only 0.03% of total reads. CNCC analysis was first implemented on the complete data set, and then on the data set that filtered out all reads mapped to AsiSI locations, to assess the ability of CNCC to detect change. To further test sensitivity, AsiSI cut sites were masked in increments of 10%, CNCC was performed as above, and output for only the − 3 shift position (the AsiSI-induced spike) was evaluated. For each 10% increment, there were 100 iterations of site masking, and only sites with more than 5 reads at the site were included in the analysis (*n* = 303). In each iteration for a 10% increment, the number of randomly picked AsiSI cut sites remained constant, while the number of reads that were masked varied as a result.

### TOP2 cleavage complex, single-nucleotide sequencing read processing and analysis

Raw fastq data (GSE136943) [37] was downloaded and aligned to the human genome (GRCh38/hg38) following the same alignment processing as our break mapping data (“Sequencing Data Analysis” above). Aligned data was then processed through our CNCC analysis (“Coverage-Normalized Cross Correlation” above) and visualized.

### BanII consensus sequence analysis

Pairs of DSB spikes separated by 4 nt (negative strand spike upstream from spike on positive strand) were identified. Of those pairs, 99.5% (*n* = 714,922) were within canonical cut sites of BanII in the reference sequence, and the remaining 0.5% of pairs (*n* = 3241) contain sequences that differ by only 1 nt from the canonical enzyme preference sequence. Sequences at all of these loci were extracted with BEDtools getfasta and the motif was found by DREAM from MEME Suit (v. 4,12.0).

### Statistical analysis

For the comparison of genomic coverage between technical duplicates of BbvI digestion and between each biological duplicate for etoposide treatment, Pearson’s correlation was calculated for the genome-wide coverage in 100-nt and 1000-nt non-overlapping windows, respectively. For the dose-dependent change in break density following etoposide treatment, Kruskal-Wallis test was performed in R (v. 3.4.3) on the normalized coverage data in each genomic region. Significant results from Kruskal-Wallis were then followed up with a Dunn test, using the Benjamini-Hochberg method, to then determine the significance of the changes between each treatment level for the specific region.

## Supplementary information

**Additional file 1: Table S1.** Reads and cut sites statistics of restriction enzyme-digested samples. **Table S2.** Sequencing and alignment statistics. **Figure S1.** Outline of genome-wide break mapping protocol. **Figure S2.** Reproducibility of genome-wide break mapping in BbvI digestion experiment. **Figure S3.** CNCC for EcoRV-digested DNA from HeLa cells. **Figure S4.** Determination of appropriate control for long-range normalized cross correlation. **Figure S5.** CNCC calculation for endogenous breaks and annotation of locations for all breaks. **Figure S6.** Evaluation of etoposide treatment on cell survival. **Figure S7.** Reproducibility of genome-wide break mapping for different etoposide treatments in GM13069. **Figure S8.** CNCC of breaks upon treatment maintains a similar trend between biological replicates for GM13069. **Figure S9.** CNCC calculated for breaks upon treatment for combined biological replicates showing shuffled control and relative CNCC signal. **Figure S10.** CNCC analysis of sequencing reads of TOP2 cleavage complex sites. **Figure S11.** Determination of consensus sequences at enzyme cut sites using CNCC.

## Data Availability

Raw data generated and reported in Figs. 1b and 3, Additional file 1: Tables S1–S2, and Figures S2, S4, S5, S7–S9, S11 (BbvI, BanII and HeaIII digestion, and TOP2 inhibition) has been deposited in the Sequence Read Archive under the accession number: PRJNA497476 (https://www.ncbi.nlm.nih.gov/sra/PRJNA497476). Publicly available data used in this study can be accessed in GEO under accession number: GSE78172 (EcoRV and AsiSI digestion experiments) and GSE136943 (Additional file 1: Figure S10).

## Abbreviations

CNCC: Coverage-normalized cross correlation
DSB: DNA double-stranded break
HR: Homologous recombination
MMEJ: Microhomology-mediated end joining
NHEJ: Non-homologous end joining
nt: Nucleotide
SSA: Single-strand annealing
TOP2: Topoisomerase II
TSS: Transcription start site
TTS: Transcription termination site
